# Prognostic value of the pre-treatment albumin-to-alkaline phosphatase ratio in patients with lower-grade glioma: a propensity score matching study

**DOI:** 10.3389/fphar.2025.1556108

**Published:** 2025-07-16

**Authors:** Xiaoming Huang, Lingjuan Li, Di Zang

**Affiliations:** ^1^ Department of Neurosurgery, Huashan Hospital, Shanghai Medical College, Fudan University, Shanghai, China; ^2^ National Center for Neurological Disorders, Shanghai, China; ^3^ Shanghai Key Laboratory of Brain Function Restoration and Neural Regeneration, Shanghai, China; ^4^ Department of Neurosurgery, China-Japan Friendship Hospital, Beijing, China

**Keywords:** AAPR, LGG, biomarker, prognosis, propensity score matching

## Abstract

**Introduction:**

The albumin-to-alkaline phosphatase ratio (AAPR) has recently emerged as a novel prognostic biomarker in various solid tumors. However, its clinical value in lower-grade glioma (LGG) remains unclear.

**Methods:**

We performed propensity score matching (PSM) to balance baseline characteristics between groups. Restricted cubic spline (RCS) analysis was used to evaluate the nonlinear relationship between AAPR and survival outcomes. Survival differences were assessed using Kaplan–Meier analysis, and both univariate and multivariate Cox regression models were applied to identify independent prognostic factors. Finally, a predictive nomogram was developed to estimate 1-, 3-, and 5-year overall survival.

**Results:**

RCS analysis revealed a nonlinear relationship between AAPR and OS (p = 0.0349). Patients were stratified by the median AAPR value (0.704), and those in the AAPR-High group (≥0.704) had significantly better OS (log-rank p = 0.0042) and progression-free survival (PFS) (log-rank p = 0.042) than those in the AAPR-Low group. AAPR showed stronger prognostic value in low-risk subgroups. Higher AAPR was significantly associated with better OS in univariate (p = 0.005, HR = 0.541, 95% CI: 0.353–0.829) and multivariate Cox analyses (p = 0.046, HR = 0.630, 95% CI: 0.400–0.993). The AAPR-based nomogram demonstrated good predictive performance for 1-, 3-, and 5-year OS, validated in the PSM cohort.

**Discussion:**

Pre-treatment AAPR is a simple, non-invasive, and effective biomarker for predicting prognosis in LGG patients, particularly those at lower clinical risk.

## 1 Introduction

Glioma is one of the most common types of brain tumors, accounting for approximately 80% of all malignant brain tumors (Ostrom et al., 2015; Lapointe et al., 2018). In accordance with the classification framework established by The Cancer Genome Atlas (TCGA), WHO Grade II and III gliomas are collectively referred to as lower-grade gliomas (LGG) (Brat et al., 2015). These tumors exhibit considerable variability in clinical behavior, which cannot be adequately predicted based solely on histological classification (Brat et al., 2015; Mair et al., 2021). Although patients with lower-grade gliomas (LGG) generally have a better prognosis than those with glioblastoma multiforme (GBM), comprehensive treatment, including surgical resection, chemotherapy, and radiotherapy, often fails to prevent treatment resistance and tumor recurrence, with more than half of LGG patients eventually progressing to highly invasive gliomas (Brat et al., 2015; Zhang et al., 2016; Hayes et al., 2018). The current WHO classification system for central nervous system tumors increasingly emphasizes molecular subtypes and prognostic biomarkers for more accurate diagnosis and treatment (Louis et al., 2021; Zhu et al., 2021). Accurate classification is essential for implementing targeted treatment strategies; however, existing methods heavily rely on complex and costly tumor tissue pathology and molecular analysis. Consequently, the identification of convenient, rapid, and cost-effective biomarkers has become critically important.

In recent years, various peripheral hematological and biochemical markers, easily obtained through minimally invasive blood tests in routine clinical practice, have gained traction for prognostic prediction across multiple cancers (Sun et al., 2014; Mei et al., 2017; Lv et al., 2018; Tan D. et al., 2018; Xu et al., 2023). Human serum albumin, the most abundant protein in plasma, is primarily produced by the liver and fulfills numerous critical biological functions. Serum albumin levels are well-established as valuable and independent biomarkers in oncology (Deme and Telekes, 2018). Beyond serum albumin levels alone, several albumin-based biomarkers, developed by combining albumin with other factors, have demonstrated prognostic value in cancer patients (Jeng et al., 2023). Serum alkaline phosphatase (ALP), an enzyme that catalyzes the hydrolysis of organic phosphate esters at alkaline pH, has also been implicated in cancer prognosis (Narayanan, 1983; Zaher et al., 2020). In 2015, Chan et al. introduced the albumin-to-alkaline phosphatase ratio (AAPR) in their study on hepatocellular carcinoma, demonstrating its strong prognostic significance (Chan et al., 2015). This finding prompted interest in applying AAPR to hepatocellular carcinoma and other malignancies. Subsequent studies have evaluated AAPR in patients with cholangiocarcinoma, breast cancer, upper urinary tract cancers, pancreatic ductal adenocarcinoma, and metastatic nasopharyngeal carcinoma, consistently identifying it as a valuable prognostic marker across these cancer types (Nie et al., 2017; Tan P. et al., 2018; Zhang F. et al., 2020; Zhang K. et al., 2020; Qu et al., 2021). However, the role of AAPR in LGG patients has yet to be explored. Therefore, investigating whether AAPR can serve as a prognostic marker in LGG patients is of significant interest.

Building on advancements in AAPR research, we conducted a retrospective study using propensity score matching, Kaplan-Meier survival analysis, Cox regression analysis, and the development of a predictive nomogram to evaluate the clinical significance of AAPR in patients with LGG, thereby offering insights into its prognostic implications for these patients.

## 2 Materials and methods

### 2.1 Study population and data

A retrospective analysis was conducted on a cohort of glioma patients who underwent surgery at the Department of Neurosurgery, Huashan Hospital, Fudan University, between 2001 and 2013. A total of 236 newly diagnosed glioma patients were included in this study. None of the patients had received any prior treatment, including chemotherapy, radiotherapy, or surgery, and all underwent at least a subtotal resection during this treatment. The extent of resection was primarily determined intraoperatively by the lead neurosurgeon under microscopy and neuronavigation guidance, with subtotal resection defined as removal of more than 90% of the tumor volume. Complete demographic information, including age, gender, postoperative pathological results, and survival endpoints (overall survival [OS] and progression-free survival [PFS]), was available for all patients. The exclusion criteria included patients with a history of autoimmune diseases, active infections, severe heart disease, chronic respiratory diseases, allergic conditions, chronic renal insufficiency, or chronic atrial fibrillation. Pathological diagnoses were confirmed by the Department of Pathology at our hospital. Based on the classification framework established by TCGA, gliomas diagnosed as WHO Grade II or III were collectively referred to as lower-grade gliomas (LGG) in this study. The study was approved by the local independent ethics committee, and written informed consent was obtained from all patients.

### 2.2 Gene detection

DNA from glioma tissues was extracted from formalin-fixed paraffin-embedded (FFPE) samples. Molecular marker detection was performed centrally using standardized protocols. Mutational hotspots in IDH1/IDH2 and the TERT promoter (TERTp) were identified through Sanger sequencing, while chromosome 1p/19q status was assessed in all patients using fluorescence *in situ* hybridization (FISH).

### 2.3 Parameters assessment

Routine blood tests were conducted within 1 week prior to surgery as part of the standard preoperative evaluation. The AAPR was calculated as the ratio of serum albumin concentration (g/L) to ALP activity (IU/L).

### 2.4 Propensity score matching analysis

To mitigate selection bias and ensure comparability between groups, Propensity Score Matching (PSM) was performed. Propensity scores were estimated using a logistic regression model, with basic characteristics included as covariates. The treatment variable of interest was the patient group stratified by AAPR. A nearest-neighbor matching algorithm with a 1:1 ratio and a caliper width of 0.02 was employed without replacement to match patients with similar propensity scores. This caliper was selected to minimize differences in propensity scores between matched individuals and ensure precise matching. After matching, 70 patients were included in each group. The balance between covariates in the two groups was assessed visually using jitter plots and quantitatively using standardized mean differences (SMDs), with an SMD less than 0.1 considered indicative of adequate balance. The PSM analysis was primarily conducted and visualized using the “MatchIt,” “mice,” “dplyr,” and “survival” R packages.

### 2.5 Restricted cubic spline analysis

Restricted cubic spline (RCS) analysis was conducted to evaluate the nonlinear relationship between AAPR and both OS and PFS in patients with LGG. The “rms,” “ggplot2,” and “survival” packages in R facilitated the RCS analysis and visualization, with a p-value of <0.1 considered statistically significant.

### 2.6 Kaplan-Meier analysis

OS and PFS were analyzed using the Kaplan-Meier method, and differences in survival times between groups were assessed using the log-rank test. The “survival” and “survminer” packages in R were utilized for performing and visualizing the Kaplan-Meier analysis.

### 2.7 Cox proportional hazards regression analysis

Univariate Cox proportional hazards regression was initially performed to evaluate the relationships between patient characteristics and survival outcomes. Clinicopathological variables with a univariate p-value <0.05 were included in a multivariate Cox proportional hazards regression model to identify independent prognostic factors for overall survival. Hazard ratios (HRs) and their corresponding 95% confidence intervals (CIs) were calculated for each variable. In this analysis, variables were included either as categorical or continuous according to their nature. Categorical variables included IDH mutation status, gender, TERT promoter mutation, 1p/19q codeletion status, tumor grade, and AAPR group (dichotomized based on the median AAPR value). Continuous variables included total protein, albumin, ALP, and age.

### 2.8 Nomogram

Nomograms were constructed to predict survival outcomes using the “regplot” package in R for visualizing predictive regression models. The “rms” package was used to build Cox proportional hazards models and generate calibration curves for evaluating model performance. The concordance index (C-index) was calculated using the “rcorr.cens” function from the “Hmisc” package to quantify the discriminative ability of the nomogram. A higher C-index indicates stronger agreement between predicted and actual outcomes, reflecting better prognostic accuracy. The 95% confidence interval of the C-index was estimated based on 1,000 bootstrap replications.

### 2.9 Time-dependent ROC curve analysis

Time-dependent ROC curves were generated using the timeROC package in R to assess the predictive accuracy of the nomogram at 1, 3, and 5 years. The linear predictor from the Cox model was applied to the PSM cohort without refitting, and AUCs were calculated to evaluate discrimination performance over time.

### 2.10 Statistical analysis and graphing

Statistical analyses and graphing were conducted using GraphPad Prism 9.0.0 and R 4.3.1 statistical software. The Shapiro-Wilk test was applied to assess the normality of continuous variables. Normally distributed data were expressed as mean ± standard deviation (SD) and compared between groups using the independent samples t-test. For non-normally distributed data, the median (interquartile range, IQR) was reported, with comparisons made using the Wilcoxon rank-sum test. Categorical data were presented as frequency (percentage), with group comparisons conducted using the χ^2^ test or Fisher’s exact test, as appropriate. All statistical tests were two-sided, and a p-value <0.05 was considered statistically significant unless otherwise specified.

## 3 Results

### 3.1 Patient characteristics

**FIGURE 1:**
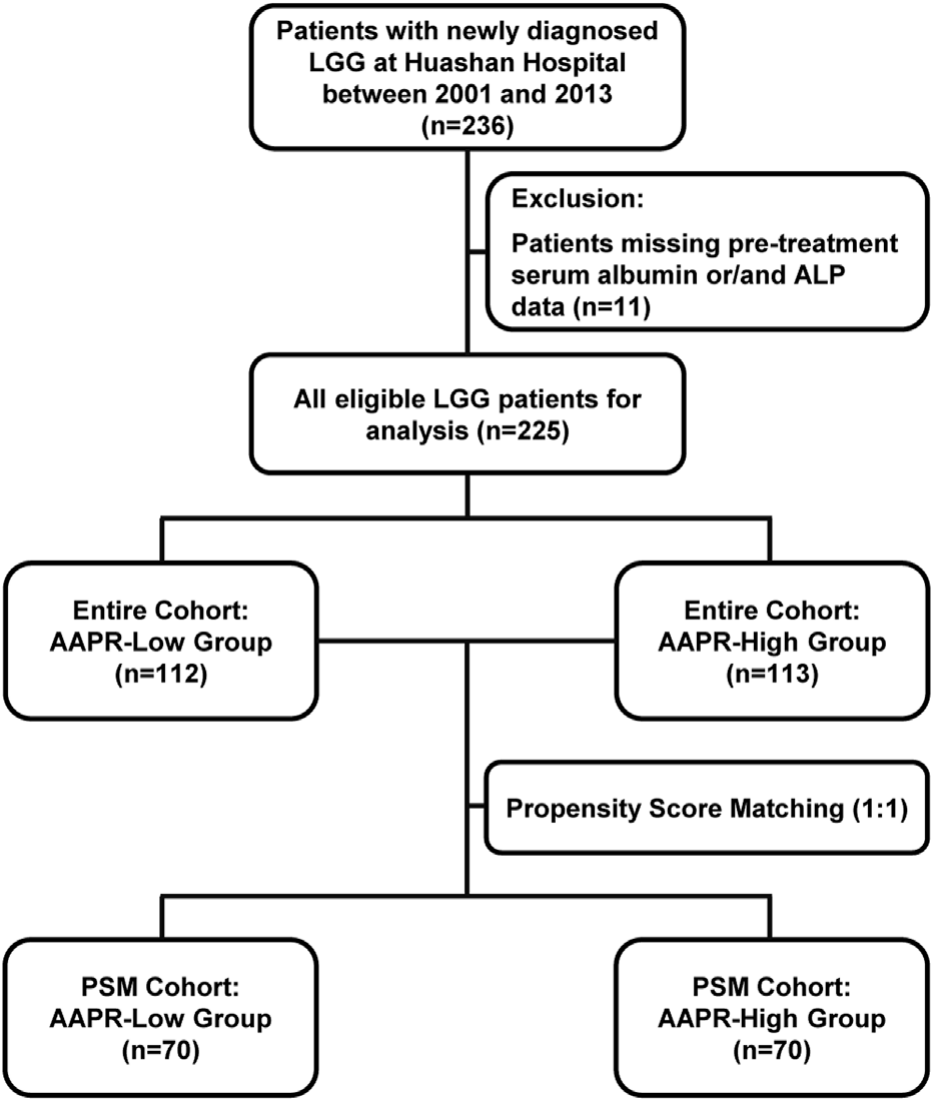
Flow chart illustrating the inclusion and exclusion of participants and the study design.

In this retrospective study, we initially included 236 patients diagnosed with LGG. However, 11 patients were excluded due to missing pre-treatment serum albumin and/or ALP data. Consequently, the final analysis comprised 225 eligible LGG patients (Figure 1). Table 1 presents the baseline characteristics of the cohort. The mean age was 42.32 years (±11.56), with 132 male patients (58.67%) and 93 female patients (41.33%). Among the patients, 142 (63.11%) had grade II gliomas, while 83 (36.89%) had grade III gliomas. Regarding histological subtype, 161 patients (71.56%) were diagnosed with astrocytoma, 64 (28.44%) with oligodendroglioma. The majority of patients (179, 79.56%) had IDH-mutant gliomas, with only 46 patients (20.44%) identified as IDH wild-type. Regarding other molecular markers, 78 patients (34.66%) had TERTp mutations, 134 patients (59.56%) did not, and data were unclear for 13 patients (5.78%). For 1p/19q co-deletion status, 64 patients (28.44%) were positive, 152 patients (67.56%) were negative, and 9 patients (4.00%) had missing data. The mean total protein level was 70.54 g/L (±5.64), the mean albumin level was 42.19 g/L (±3.20), and the median alkaline ALP level was 59 IU/L (IQR: 49–74). The cohort was divided into two groups based on the median AAPR value: the AAPR-Low group (n = 112) with AAPR <0.704, and the AAPR-High group (n = 113) with AAPR ≥0.704. Most baseline characteristics were comparable between the two groups. However, the proportion of patients with 1p/19q co-deletion was significantly higher in the AAPR-High group (37.72%) than in the AAPR-Low group (19.27%) (p = 0.003). Similarly, the distribution of pathological subtypes was unbalanced between groups, with a significantly higher proportion of oligodendrogliomas in the AAPR-High group (38.05%) compared to the AAPR-Low group (18.75%) (p = 0.002). Serum total protein and albumin levels were largely comparable, with the primary difference observed in ALP levels. The median ALP level in the AAPR-Low group was 74 IU/L (IQR: 65–82), while the AAPR-High group had a significantly lower median ALP level of 49 IU/L (IQR: 40–54), a difference that was statistically significant (p < 0.0001).

**TABLE 1:** Baseline characteristics of LGG patients in entire and PSM cohorts by AAPR status.

	Entire cohort				PSM cohort			
Characteristic	Total (n = 225)	AAPR-low (n = 112)	AAPR-high (n = 113)	*P*_value	Total (n = 140)	AAPR-low (n = 70)	AAPR-high (n = 70)	*P*_value
Age (mean ± sd)	42.32 ± 11.56	42.44 ± 11.91	42.19 ± 11.27	0.875	41.04 ± 10.00	40.77 ± 10.44	41.31 ± 9.68	0.750
Age (median Range)	41.00 (5.00–79.00)	41.00 (5.00–79.00)	41.00 (16.00–71.00)	0.774	40.00 (7.00–79.00)	39.50 (7.00–79.00)	41.00 (16.00–68.00)	0.532
Gender								
Male	132 (58.67%)	62 (55.36%)	70 (61.95%)	0.316	84 (60.00%)	41 (58.57%)	43 (61.43%)	0.730
Female	93 (41.33%)	50 (44.64%)	43 (38.05%)		56 (40.00%)	29 (41.43%)	27 (38.57%)	
Pathology subtype								
Astrocytoma	161 (71.56%)	91 (81.25%)	70 (61.95%)	0.0022	103 (73.57%)	51 72.86 (%)	52 (74.29%)	1.00
Oligodendroglioma	64 (28.44%)	21 (18.75%)	43 (38.05%)		37 (26.43%)	19 (27.14%)	18 (25.71%)	
WHO Grade								
Grade II	142 (63.11%)	65 (58.04%)	77 (68.14%)	0.116	94 (67.14%)	48 (68.57%)	46 (65.71%)	0.719
Grade III	83 (36.89%)	47 (41.96%)	36 (31.86%)		46 (32.86%)	22 (31.43%)	24 (34.29%)	
IDH Mutation								
Mutation	179 (79.56%)	88 (78.57%)	91 (80.53%)	0.716	115 (82.14%)	57 (81.43%)	58 (82.86%)	0.825
Wild	46 (20.44%)	24 (21.43%)	22 (19.47%)		25 (17.86%)	13 (18.57%)	12 (17.14%)	
TERTp Mutation								
Mutation	78 (34.66%)	39 (34.21%)	39 (35.14%)	0.834	47 (33.57%)	25 (35.71%)	22 (31.43%)	0.631
Wild	134 (59.56%)	69 (60.53%)	65 (58.56%)		86 (61.43%)	42 (60.00%)	44 (62.86%)	
NA	13 (5.78%)	4 (3.51%)	9 (8.11%)		7 (5.00%)	3 (4.29%)	4 (5.71%)	
1p/19q Codeletion								
Codeletion	64 (28.44%)	21 (19.27%)	43 (37.72%)	0.003	37 (26.40%)	19 (27.14%)	18 (25.71%)	0.725
Non-Codeletion	152 (67.56%)	83 (76.15%)	69 (60.53%)		98 (70.00%)	47 (67.14%)	51 (72.86%)	
NA	9 (4.00%)	8 (7.34%)	1 (0.88%)		5 (3.57%)	4 (5.71%)	1 (1.43%)	
Total Protein (mean ± sd, g/L)	70.54 ± 5.64	70.56 ± 5.83	70.51 ± 5.46	0.948	70.70 ± 5.48	70.56 ± 6.04	70.84 ± 4.89	0.759
Albumin (mean ± sd, g/L)	42.19 ± 3.20	41.85 ± 3.29	42.52 ± 3.09	0.115	42.16 ± 3.22	41.63 ± 3.36	42.70 ± 2.99	0.048
ALP (median IQR, IU/L)	59.00 (49.00–74.00)	74.00 (65.00–82.00)	49.00 (40.00–54.00)	<0.001	58.00 (49.00–72.00)	74.00 (64.00–80.00)	49.00 (41.25–54.75)	<0.001

**FIGURE 2:**
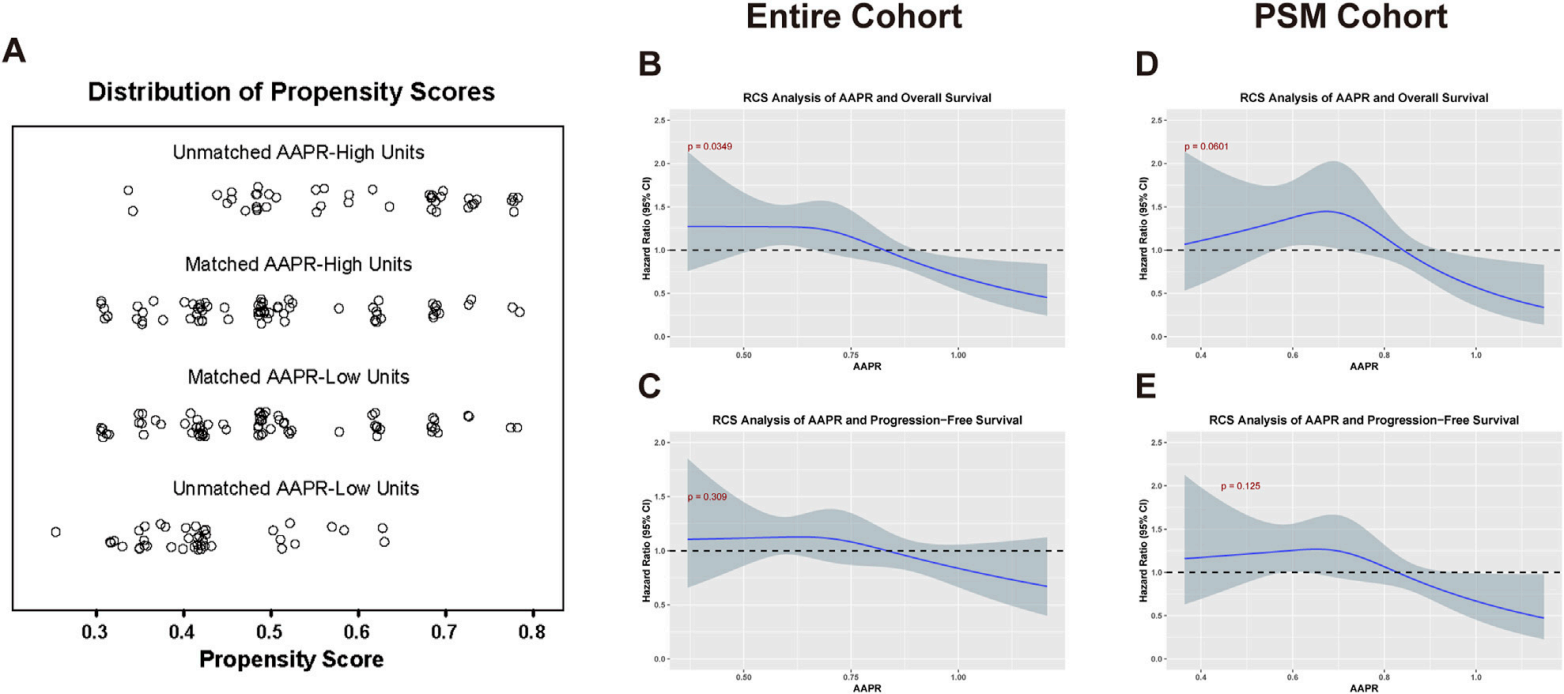
Analysis of AAPR in relation to OS and PFS in the entire and PSM cohorts. **(A)** Displays the distribution of propensity scores for matched and unmatched units in the AAPR-High and AAPR-Low groups. **(B,C)** illustrate the RCS analysis of the AAPR in relation to OS (p = 0.0349) and PFS (p = 0.125), respectively, within the entire cohort. **(D,E)** Show the corresponding RCS analysis for OS (p = 0.0601) and PFS (p = 0.309) in the PSM cohort. In the RCS analysis, a p-value threshold of <0.1 was considered statistically significant.

After stratification by the median AAPR value, the baseline characteristics of the two groups were not fully balanced. Notably, the 1p/19q co-deletion status and pathology subtype differed significantly between groups (Table 1). Given the critical role of 1p/19q co-deletion in the prognosis of LGG patients, we addressed this imbalance using propensity score matching (Figure 1). After matching, 70 patients were included in both the AAPR-High and AAPR-Low groups (Figure 2A), resulting in balanced baseline characteristics (Table 1). The cohort after matching is referred to as the PSM cohort, while the cohort before matching is referred to as the entire cohort. Importantly, the PSM cohort was used to validate the robustness and generalizability of findings initially derived from the entire cohort, thereby strengthening the reliability of our conclusions.

### 3.2 Correlation between AAPR and prognosis in patients with LGG

To investigate the impact of AAPR on the prognosis of LGG patients, we performed restricted cubic spline (RCS) analysis to evaluate potential nonlinear associations between AAPR and patient outcomes. In the entire cohort, a statistically significant nonlinear relationship was observed between AAPR and OS (p = 0.0349) (Figure 2B). In the PSM cohort, the analysis suggested a potential nonlinear pattern with a p-value approaching statistical significance (p = 0.0601) (Figure 2D). No significant nonlinear association was found between AAPR and PFS in either cohort (Figures 2C,E). Overall, the RCS curves demonstrated that higher AAPR levels were consistently associated with lower hazard ratios, suggesting a protective effect of elevated AAPR on survival outcomes.

### 3.3 Prognostic value of AAPR

**FIGURE 3:**
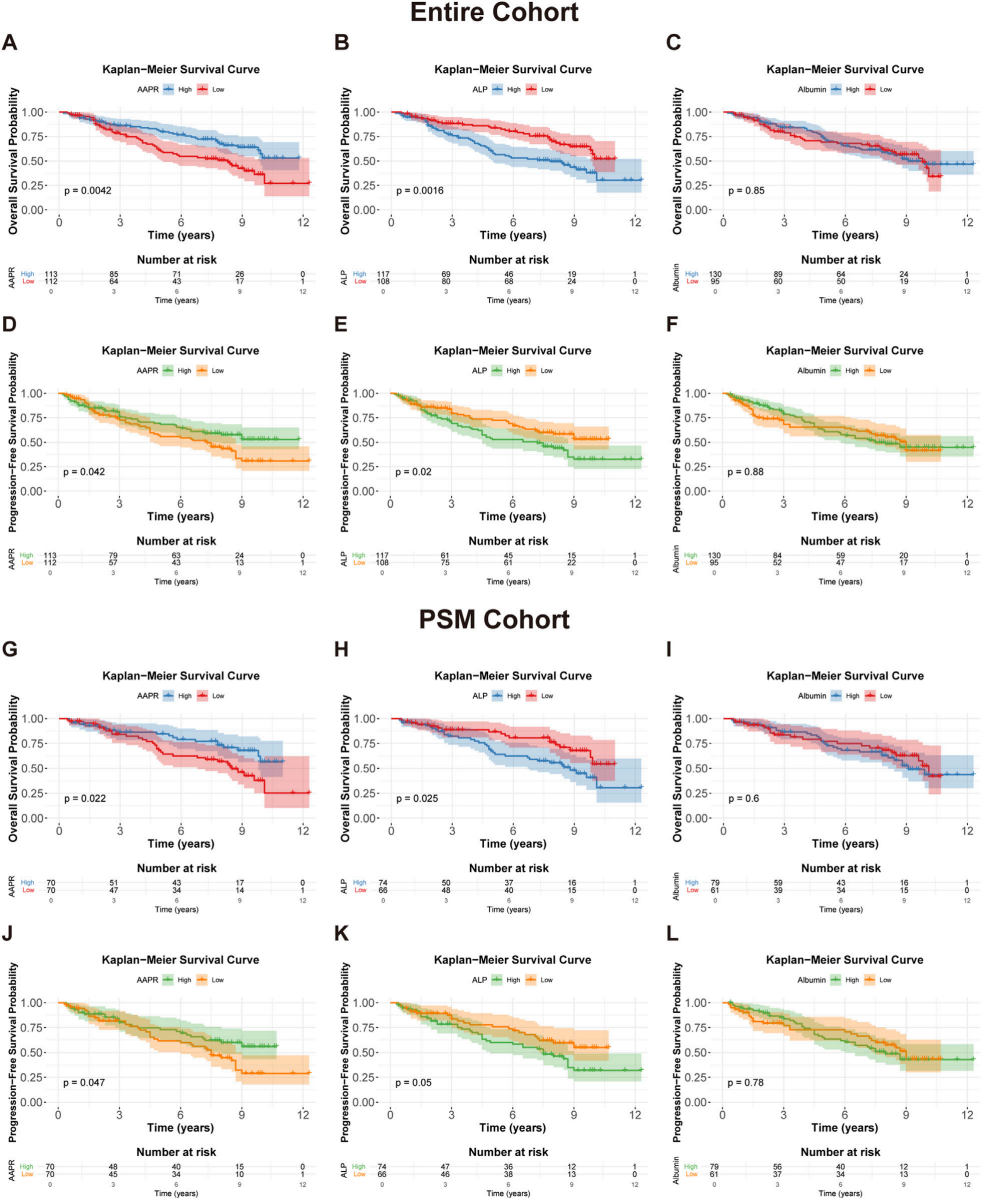
Kaplan-Meier survival curves for OS and PFS in the entire cohort and PSM cohort. **(A,D)** Kaplan-Meier survival curves for OS (p = 0.0042) and PFS (p = 0.042) in the entire cohort, stratified by AAPR. **(B,E)** show OS (p = 0.0016) and PFS (p = 0.02) curves in the entire cohort, stratified by median serum ALP levels. **(C,F)** display OS (p = 0.85) and PFS (p = 0.88) curves in the entire cohort, stratified by median serum albumin levels. **(G,J)** Kaplan-Meier survival curves for OS (p = 0.022) and PFS (p = 0.047) in the PSM cohort, stratified by AAPR. **(H,K)** present OS (p = 0.025) and PFS (p = 0.05) curves in the PSM cohort, stratified by median serum ALP levels. **(I,L)** show OS (p = 0.6) and PFS (p = 0.78) curves in the PSM cohort, stratified by median serum albumin levels.

To evaluate the impact of the AAPR on the prognosis of patients with LGG, we conducted Kaplan-Meier survival analysis based on AAPR groupings. The results demonstrated that both OS (p = 0.0042) (Figure 3A) and PFS (p = 0.042) (Figure 3D) were significantly lower in the AAPR-Low group compared to the AAPR-High group. Additionally, analysis of the PSM cohort confirmed significant differences in AAPR’s effect on OS (p = 0.022) (Figure 3G) and PFS (p = 0.047) (Figure 3J) among LGG patients. These findings align with our earlier RCS analysis, reinforcing the conclusion that elevated AAPR serves as a favorable prognostic marker in LGG patients.

To further assess the prognostic value of AAPR in LGG, we conducted stratified analyses based on relevant clinical factors. Prior to stratification, we confirmed the prognostic significance of these factors within our cohort. In the entire cohort, WHO Grade II, IDH mutations, and 1p/19q co-deletion were significantly associated with better outcomes in LGG patients, while TERTp mutations were not correlated with prognosis (Supplementary Figure S1A–H). In the PSM cohort, WHO Grade II, IDH mutations, and 1p/19q co-deletion remained significantly linked to improved prognosis, and TERTp mutations became significantly associated with better outcomes (Supplementary Figure S1I–P). The prognostic characteristics of these factors were generally consistent with previously reported findings.

**FIGURE 4:**
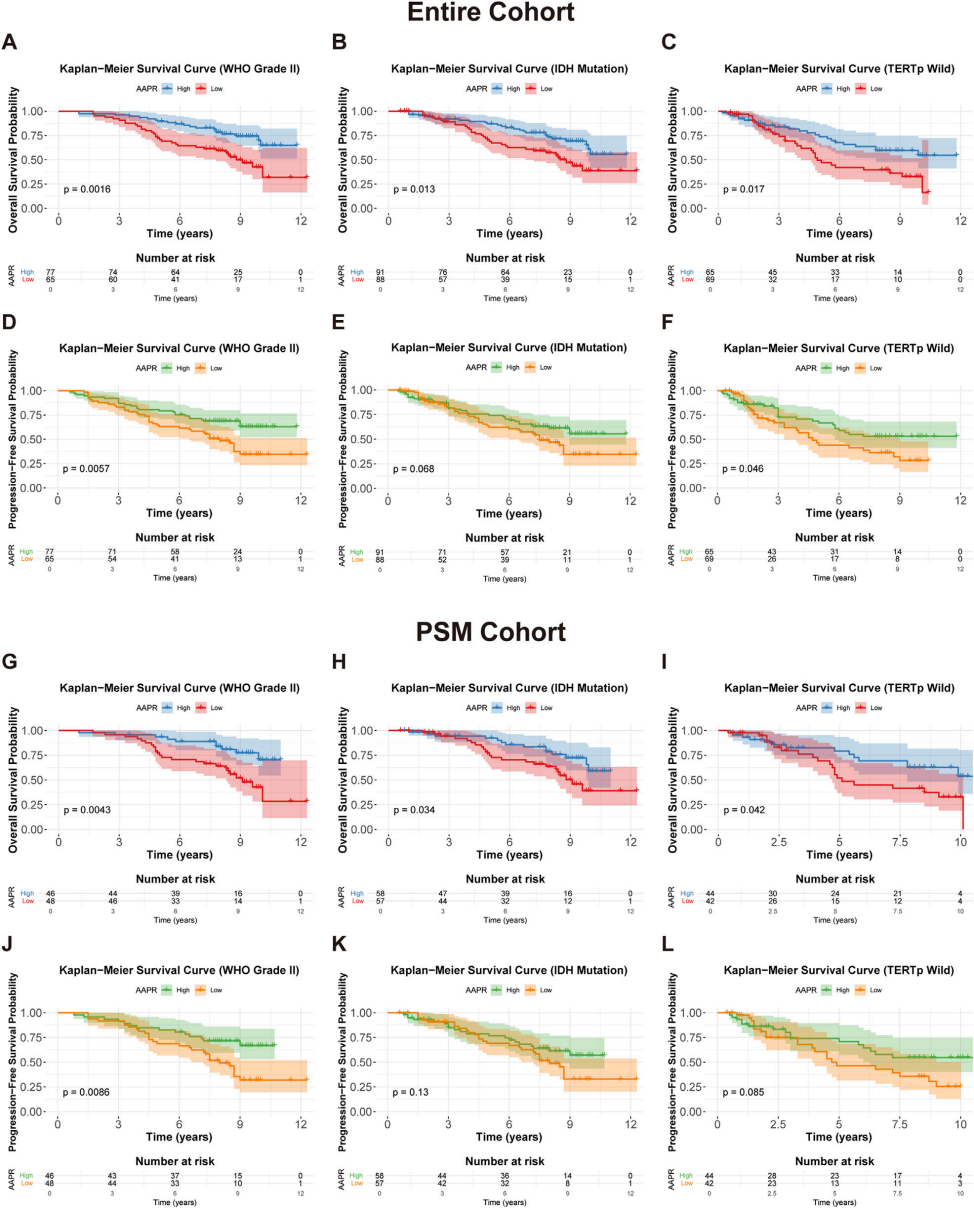
Kaplan-Meier survival curves for OS and PFS stratified by AAPR in different clinical subgroups. **(A,D)** Show OS and PFS curves for WHO Grade II patients in the entire cohort, stratified by AAPR (p = 0.0016 and p = 0.0057, respectively). **(B,E)** display OS and PFS curves for IDH mutation patients in the entire cohort, stratified by AAPR (p = 0.013 and p = 0.068, respectively). **(C,F)** illustrate OS and PFS for TERTp wild-type patients in the entire cohort, stratified by AAPR (p = 0.017 and p = 0.046, respectively). **(G,J)** represent OS and PFS curves for WHO Grade II patients in the PSM cohort, stratified by AAPR (p = 0.0043 and p = 0.0086, respectively). **(H,K)** show OS and PFS curves for IDH mutation patients in the PSM cohort, stratified by AAPR (p = 0.0034 and p = 0.13, respectively). **(I,L)** depict OS and PFS curves for TERTp wild-type patients in the PSM cohort, stratified by AAPR (p = 0.042 and p = 0.085, respectively).

Our analysis revealed that in the entire cohort, high AAPR was a strong predictor of favorable OS and PFS in WHO Grade II LGG (Figures 4A,D), IDH-mutant tumors (Figures 4B,E), and TERTp wild-type tumors (Figures 4C,F). Similar results were observed in the PSM cohort (Figures 4G–J). Although the Kaplan-Meier analysis based on PFS for IDH-mutant (Figure 4K) and TERTp wild-type (Figure 4L) patients in the PSM cohort did not reach statistical significance, the overall trend was consistent with that of the entire cohort. Conversely, AAPR did not show significant prognostic value in WHO Grade III LGG (Supplementary Figures S2C, H), IDH wild-type tumors (Supplementary Figures S2D,I), or TERTp-mutated tumors (Supplementary Figures S2E,J). AAPR also showed no significant prognostic value in the stratified analysis based on 1p/19q status (Supplementary Figures S2A,B,F,G). These results were consistent in the PSM cohort (Supplementary Figures S2K–T), suggesting that AAPR may have greater predictive value in low-risk LGG patients.

Additionally, we performed a Kaplan–Meier survival analysis stratified by the median values of serum ALP (59 IU/L) and albumin (42 g/L). This analysis revealed that high ALP was associated with significantly lower OS (p = 0.0016) (Figure 3B) and PFS (p = 0.02) (Figure 3E) in LGG patients, while serum albumin did not show statistical significance in the survival analysis (Figures 3C,F). These findings were further validated in the PSM cohort (Figures 3H,I,K,L). It appears that AAPR primarily affects patient prognosis through variations in ALP levels. This is consistent with the observed differences in ALP distribution between the two groups, as the median ALP value in the AAPR-Low group was significantly higher than in the AAPR-High group (Table 1). In contrast, the difference in serum albumin between the two groups was less pronounced (Table 1).

### 3.4 AAPR as an independent prognostic marker for LGG patients

**FIGURE 5:**
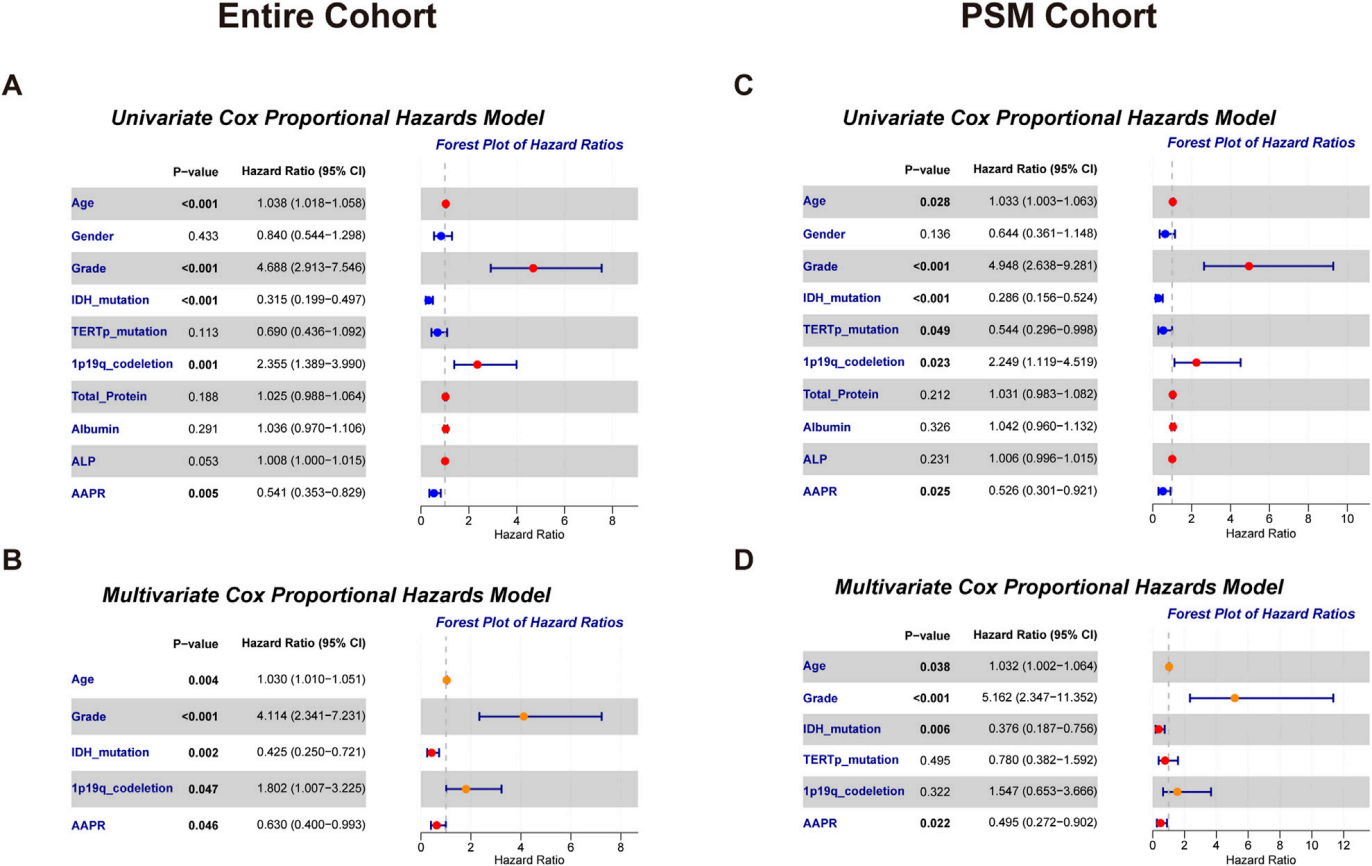
Forest plots from univariate and multivariate Cox proportional hazards models for overall survival in the entire cohort and PSM cohort. **(A)** Univariate model results highlighting key predictors in the entire cohort. **(B)** Multivariate model results showing adjusted effects in the entire cohort. **(C)** Univariate model findings in the PSM cohort. **(D)** Multivariate model results for the PSM cohort, emphasizing the impact of selected variables.

To assess the prognostic value of AAPR more accurately, we incorporated various clinicopathological factors and applied Cox proportional hazards models for OS analysis. In the entire cohort, univariate Cox analysis identified several significant prognostic factors for LGG: age (P < 0.001, HR = 1.038, 95% CI: 1.018–1.058), WHO tumor grade (P < 0.001, HR = 4.688, 95% CI: 2.913–7.546), IDH mutation status (P < 0.001, HR = 0.315, 95% CI: 0.199–0.497), 1p/19q co-deletion (P = 0.001, HR = 2.355, 95% CI: 1.389–3.990), and AAPR (P = 0.005, HR = 0.541, 95% CI: 0.353–0.829) (Figure 5A). After adjusting for these variables in multivariate Cox analysis, AAPR (P = 0.046, HR = 0.630, 95% CI: 0.400–0.993) remained a significant prognostic factor (Figure 5B). Furthermore, in both univariate and multivariate Cox models, AAPR consistently acted as a protective factor (HR < 1), reinforcing findings that elevated AAPR is associated with favorable prognosis in LGG patients.

In the PSM cohort, univariate Cox analysis similarly identified age (P = 0.028, HR = 1.033, 95% CI: 1.003–1.063), WHO tumor grade (P < 0.001, HR = 4.948, 95% CI: 2.638–9.281), IDH mutation status (P < 0.001, HR = 0.286, 95% CI: 0.156–0.524), TERTp mutation status (P = 0.049, HR = 0.544, 95% CI: 0.296–0.998), 1p/19q co-deletion (P = 0.023, HR = 2.249, 95% CI: 1.119–4.519), and AAPR (P = 0.025, HR = 0.526, 95% CI: 0.301–0.921) as significant prognostic factors (Figure 5C). Multivariate Cox analysis further confirmed AAPR (P = 0.022, HR = 0.495, 95% CI: 0.272–0.902) as a significant independent prognostic factor in LGG patients (Figure 5D), further validating the prognostic value of AAPR.

Notably, while serum albumin and ALP were not independent risk factors in the Cox proportional hazards model (Figures 5A,C), combining these markers into the AAPR provided a more sensitive predictor of patient prognosis.

### 3.5 Nomogram construction and validation for prognostic prediction in LGG patients

**FIGURE 6:**
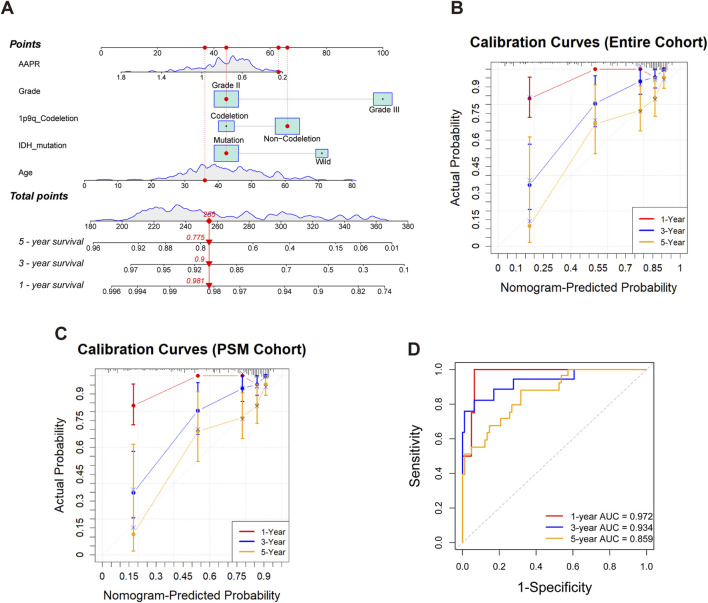
Construction and validation of the nomogram for predicting 1-, 3-, and 5-year survival in LGG patients. **(A)** Nomogram for the entire cohort integrating AAPR, tumor grade, 1p/19q codeletion status, IDH mutation, and age. Each variable corresponds to a point scale, and the total score is mapped to estimated survival probabilities at 1, 3, and 5 years. **(B)** Calibration curves for the entire cohort, illustrating good concordance between predicted and observed survival at 1, 3, and 5 years. **(C)** Calibration curves for the PSM cohort, demonstrating good agreement between predicted and observed survival in the matched population. **(D)** Time-dependent ROC curves for the PSM cohort at 1, 3, and 5 years, demonstrating the discriminative performance of the nomogram.

As shown in Figure 6A, a nomogram was constructed for the entire cohort by integrating AAPR with independent clinical risk factors. Each variable was assigned a corresponding point on the top scale. The total score, ranging from 180 to 380, is used to estimate 1-, 3-, and 5-year survival probabilities shown on the lower scales. A higher total score indicates a poorer prognosis. For example, a patient with a total score of 285 has estimated survival probabilities of 98.1% at 1 year, 90.0% at 3 years, and 77.5% at 5 years. The calibration curve for the entire cohort (Figure 6B) demonstrated excellent agreement between predicted and observed survival, indicating high predictive accuracy. The nomogram yielded a C-index of 0.756 based on 1,000 bootstrap resamples (95% CI: 0.702–0.811), confirming its discriminative ability.

To further validate the model, we applied the same nomogram—without refitting—to the PSM cohort. Calibration performance remained good (Figure 6C), with a C-index of 0.753 (95% CI: 0.669–0.828), indicating robust generalizability in a well-balanced subset. Time-dependent ROC analysis based on the PSM cohort (Figure 6D) showed strong prognostic discrimination, with AUCs of 0.972, 0.934, and 0.859 for predicting 1-, 3-, and 5-year survival, respectively.

In conclusion, the nomogram demonstrated excellent performance in both the entire and PSM cohorts, supporting its potential for clinical application in prognostic prediction for LGG patients.

## 4 Discussion

The AAPR is a novel, convenient, cost-effective, and non-invasive indicator derived from serum albumin and ALP levels, offering valuable insights into systemic inflammation and nutritional status (Li et al., 2020). Since its introduction by Chan et al. (2015) as a predictor of surgical outcomes in hepatocellular carcinoma, AAPR has gained recognition as a promising prognostic indicator across a variety of malignancies (Sun et al., 2014; Mei et al., 2017; Lv et al., 2018; Tan D. et al., 2018; Xu et al., 2023). Recent literature reviews suggest that AAPR significantly influences the prognosis of solid tumors, irrespective of their origin (Tian et al., 2020; An et al., 2021). However, its clinical significance in LGG patients remains unexplored.

In this retrospective study, we found that pre-treatment AAPR was significantly associated with the prognosis of LGG patients, particularly in relation to OS, as demonstrated through nonlinear correlation analysis. Kaplan-Meier survival analysis indicated that a low AAPR was strongly correlated with poor patient prognosis, consistent with findings from previous studies in other tumor types. Subgroup analysis further revealed that a low AAPR was significantly associated with poor prognosis in patients with WHO Grade II tumors, IDH mutations, and TERT promoter wild-type LGG. However, this association was not observed in patients with WHO Grade III tumors, IDH wild-type, or TERTp mutations, suggesting that AAPR may be more sensitive in predicting outcomes for lower-risk LGG patients. Additionally, univariate and multivariate Cox regression analyses confirmed that AAPR is an independent prognostic factor, even after adjusting for age, WHO grade, and molecular pathological markers. Furthermore, we developed a nomogram to evaluate the prognosis of LGG patients by integrating AAPR with other independent clinical risk factors. To minimize potential confounding effects and balance the comparison groups, propensity score matching (PSM) was applied, resulting in 70 matched pairs. The findings from the entire cohort were subsequently validated in the PSM cohort. The consistent prognostic value of AAPR across both the entire and matched cohorts strengthens its potential as a reliable biomarker for LGG prognosis.

Serum albumin serves as a critical nutritional indicator and is integral to various biochemical processes, including DNA replication, cell growth, and enhancing the antioxidant effects of anticancer agents (Arroyo et al., 2014). Hypoalbuminemia, a condition characterized by low serum albumin levels, not only indicates malnutrition but also reflects a sustained systemic inflammatory response, which can compromise anti-tumor immunity (Calder and Kew, 2002; Mantovani et al., 2008; Li et al., 2019). Additionally, numerous studies have demonstrated that pretreatment serum albumin is a significant prognostic marker for tumors, with low serum albumin levels being strongly associated with poor outcomes in cancer patients (Chen et al., 2016; Liu and Wang, 2020; Guven et al., 2022).

ALP is a phosphomonoester hydrolase that facilitates the hydrolysis and transfer of phosphate groups under alkaline conditions (Vimalraj, 2020). It is commonly used in routine clinical practice to assess liver function. Emerging evidence suggests that ALP is also an important prognostic marker for cancer patients (Ren et al., 2015; Li et al., 2018; Jiang et al., 2023). Elevated ALP levels reliably indicate oxidative stress—a byproduct of inflammation that generates reactive oxygen species, leading to DNA, protein, and lipid damage, which promotes mutagenic activity, cancer progression, and poorer disease outcomes (Mantovani et al., 2008; López-Posadas et al., 2011; Martinez-Useros et al., 2017; Pu et al., 2017).

Although Kaplan-Meier analysis indicated that elevated ALP levels are associated with poor prognosis in patients with LGG, Cox regression analysis failed to designate ALP as an independent prognostic factor. In contrast, our analysis identified the AAPR as a significant independent prognostic factor. Even after adjusting for potential confounders, AAPR maintained its prognostic significance, a finding further validated in the PSM cohort. Single biomarkers can be influenced by various confounding factors and may not fully capture the complex biological processes underlying tumor progression. Composite indices that integrate multiple relevant biomarkers can provide a more accurate and sensitive assessment of prognosis. The superior prognostic performance of AAPR over ALP alone may be attributed to its composite nature, as it integrates both serum albumin and ALP levels into a single index. By combining these two biomarkers, AAPR offers a more comprehensive assessment of a patient’s physiological and pathological state, capturing the interplay between nutrition, inflammation, and tumor biology.

Moreover, AAPR proved particularly effective in predicting outcomes for lower-risk LGG patients, suggesting its potential utility in identifying higher-risk individuals within this subgroup. This specificity enhances the value of AAPR as a prognostic tool, enabling more precise risk stratification and potentially guiding clinical decision-making regarding treatment intensity and follow-up care for different patient groups.

However, our study is not without limitations. Being a retrospective analysis from a single institution, there is a potential for selection bias and limitations in generalizability. Although we applied PSM to minimize confounding, residual confounders may still persist. One limitation is the lack of complete data on postoperative adjuvant therapies and MGMT promoter methylation status, both of which may have influenced survival outcomes. However, the impact of these factors may have been partially mitigated by PSM and the relatively balanced baseline characteristics between groups. Additionally, the sample size, particularly in subgroup analyses, may limit the statistical power of our findings. While we were unable to validate the model in an independent cohort, we performed internal validation using the PSM cohort, which further supported the robustness of our results. Future prospective, multicenter studies with larger cohorts are needed to externally validate our findings and further investigate the mechanisms by which AAPR influences prognosis in LGG patients.

## 5 Conclusion

In conclusion, our study demonstrates that AAPR is a more sensitive and independent prognostic marker for patients with LGG compared to ALP alone. The integration of serum albumin and ALP levels into a single index provides a more holistic view of the patient’s health status, reflecting both nutritional and inflammatory conditions that are pivotal in cancer progression. Incorporating AAPR into clinical practice could enhance prognostic assessments, aid in risk stratification, and guide more tailored therapeutic strategies, ultimately improving patient outcomes in LGG.

## Data Availability

The original contributions presented in this study are included in the article. Further inquiries regarding data access can be directed to the corresponding authors.

## Supplementary material

SUPPLEMENTARY FIGURE S1 Kaplan-Meier survival curves for OS and PFS stratified by various clinical and molecular factors in the entire cohort and PSM cohort. **(A,E)** Show OS and PFS curves stratified by WHO grade in the entire cohort, with both showing significant differences (p < 0.0001 for OS and PFS). **(B,F)** Display OS and PFS curves stratified by IDH mutation status in the entire cohort, with significant survival advantages for IDH-mutant patients (p < 0.0001 for both OS and PFS). **(C,G)** Present OS and PFS curves based on 1p/19q codeletion status in the entire cohort, with significant differences in both OS (p = 0.001) and PFS (p = 0.005). **(D,H)** Illustrate OS and PFS curves stratified by TERTp mutation status in the entire cohort, showing no significant differences in OS (p = 0.11) and PFS (p = 0.21). **(I,M)** Show OS and PFS curves stratified by WHO grade in the PSM cohort, both showing highly significant differences (p < 0.0001 for OS and PFS). **(J,N)** Depict OS and PFS curves based on IDH mutation status in the PSM cohort, with significant differences in OS (p < 0.0001) and PFS (p = 0.0012). **(K,O)** Present OS and PFS curves according to 1p/19q codeletion status in the PSM cohort, with significant differences in OS (p = 0.019) but no significant difference in PFS (p = 0.066). **(L,P)** Show OS and PFS curves stratified by TERTp mutation status in the PSM cohort, with a significant difference in OS (p = 0.046) but no significant difference in PFS (p = 0.21).

SUPPLEMENTARY FIGURE S2 Kaplan-Meier survival curves for OS and PFS stratified by AAPR across different clinical and molecular subgroups in the entire cohort and PSM cohort. **(A–E)** OS curves for patients in the entire cohort stratified by AAPR for the following subtypes: **(A)** 1p/19q codeletion (p = 0.39), **(B)** 1p/19q non-codeletion (p = 0.12), **(C)** WHO Grade III (p = 0.6), **(D)** IDH wild-type (p = 0.2), and **(E)** TERTp mutation (p = 0.074). **(F–J)** Display PFS curves for patients in the entire cohort stratified by AAPR for the following subtypes: **(F)** 1p/19q codeletion (p = 0.28), **(G)** 1p/19q non-codeletion (p = 0.63), **(H)** WHO Grade III (p = 0.54), **(I)** IDH wild-type (p = 0.4), and **(J)** TERTp mutation (p = 0.36). **(K–O)** Show OS curves for patients in the PSM cohort stratified by AAPR for the following subtypes: **(K)** 1p/19q codeletion (p = 0.11), **(L)** 1p/19q non-codeletion (p = 0.13), **(M)** WHO Grade III (p = 0.52), **(N)** IDH wild-type (p = 0.27), and **(O)** TERTp mutation (p = 0.15). **(P–T)** Show PFS curves for patients in the PSM cohort stratified by AAPR for the following subtypes: **(P)** 1p/19q codeletion (p = 0.39), **(Q)** 1p/19q non-codeletion (p = 0.14), **(R)** WHO Grade III (p = 0.72), **(S)** IDH wild-type (p = 0.2), and **(T)** TERTp mutation (p = 0.31).
