# Supplementary figures and images for: Prognostic value of the pre-treatment albumin-to-alkaline phosphatase ratio in patients with lower-grade glioma: a propensity score matching study
Source: Front Pharmacol. 2025 Jul 16;16:1556108. doi: 10.3389/fphar.2025.1556108 (PMC12307405; doi:10.3389/fphar.2025.1556108)

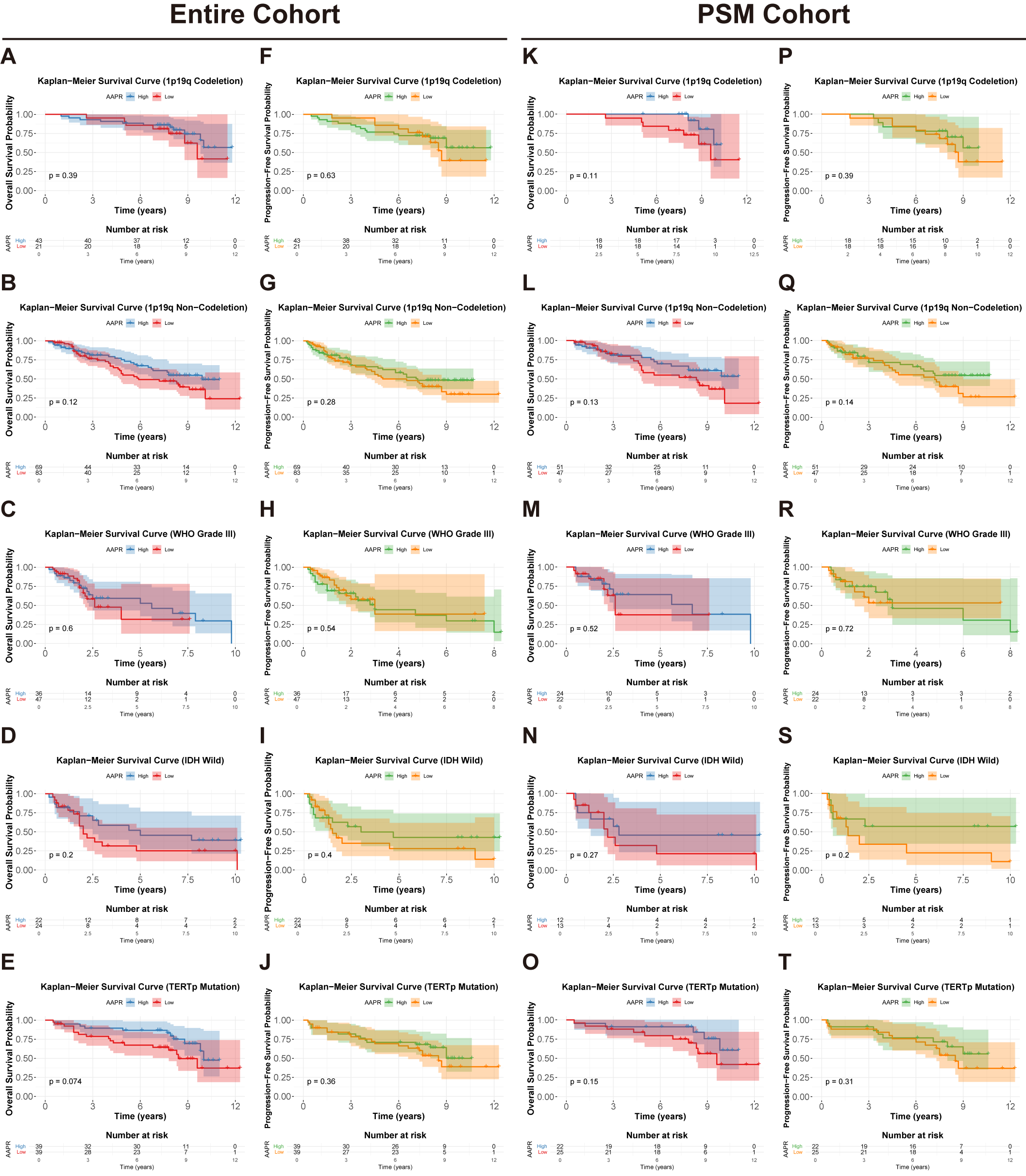

Supplement: Supplementary file 1 [file Image2.tif]

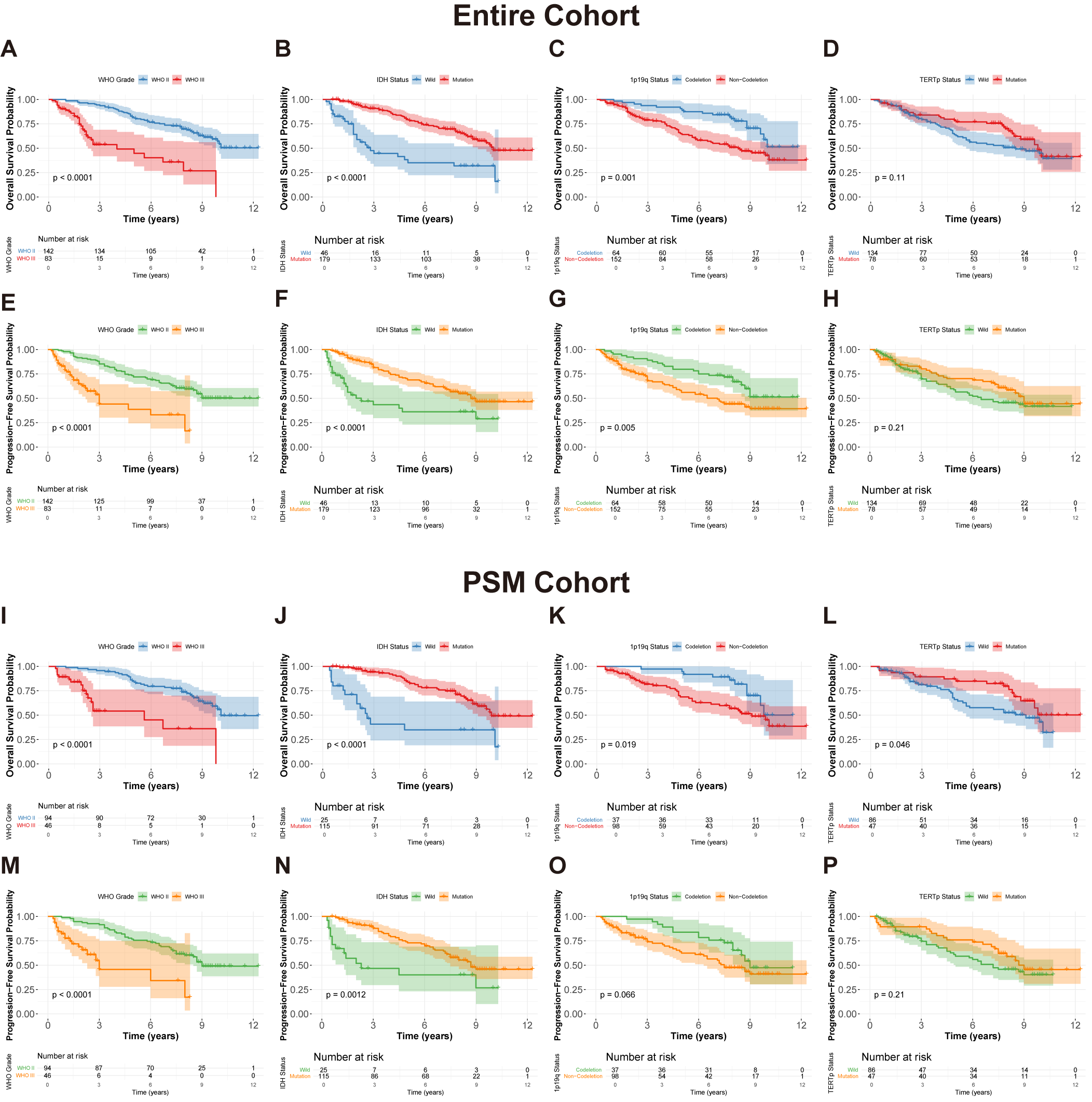

Supplement: Supplementary file 2 [file Image1.tif]
